# Application of a preclinical 18-gene classifier to patients with locally advanced HNSCC

**DOI:** 10.1016/j.ctro.2025.101067

**Published:** 2025-11-05

**Authors:** Steffen Löck, Lydia Koi, Kristin Gurtner, Fabian Lohaus, Max Kemper, Dominik Haim, Inge Tinhofer, Goda Kalinauskaite, Martin Stuschke, Maximilian Fleischmann, Claus Rödel, Anca-Ligia Grosu, Jürgen Debus, Claus Belka, Stephanie Combs, Simon Boeke, Gustavo Baretton, Michael Baumann, Mechthild Krause, Annett Linge

**Affiliations:** a German Cancer Consortium (DKTK), Partner Site Dresden, and German Cancer Research Center (DKFZ), Heidelberg, Germany; b German Cancer Consortium (DKTK), Partner Site Berlin, and German Cancer Research Center (DKFZ), Heidelberg, Germany; c German Cancer Consortium (DKTK), Partner Site Essen, and German Cancer Research Center (DKFZ), Heidelberg, Germany; d German Cancer Consortium (DKTK), Partner Site Frankfurt, and German Cancer Research Center (DKFZ), Heidelberg, Germany; e German Cancer Consortium (DKTK), Partner Site Freiburg, and German Cancer Research Center (DKFZ), Heidelberg, Germany; f German Cancer Consortium (DKTK), Partner Site Heidelberg, and German Cancer Research Center (DKFZ), Heidelberg, Germany; g German Cancer Consortium (DKTK), Partner Site Munich, and German Cancer Research Center (DKFZ), Heidelberg, Germany; h German Cancer Consortium (DKTK), Partner Site Tübingen, and German Cancer Research Center (DKFZ), Heidelberg, Germany; i OncoRay – National Center for Radiation Research in Oncology, Faculty of Medicine and University Hospital Carl Gustav Carus, TUD Dresden University of Technology, Helmholtz-Zentrum Dresden-Rossendorf, Dresden, Germany; j Helmholtz-Zentrum Dresden - Rossendorf, Institute of Radiooncology – OncoRay, Dresden, Germany; k National Center for Tumor Diseases (NCT), NCT/UCC Dresden, a Partnership between DKFZ, Faculty of Medicine and University Hospital Carl Gustav Carus, TUD Dresden University of Technology, and Helmholtz-Zentrum Dresden-Rossendorf (HZDR), Germany; l Department of Radiotherapy and Radiation Oncology, Faculty of Medicine and University Hospital Carl Gustav Carus, TUD Dresden University of Technology, Dresden, Germany; m Department of Otorhinolaryngology, Faculty of Medicine and University Hospital Carl Gustav Carus, Technische Universität Dresden, Germany; n Department of Oral and Maxillofacial Surgery, Faculty of Medicine and University Hospital Carl Gustav Carus, Technische Universität Dresden, Germany; o Department of Radiooncology and Radiotherapy, Charité, University Medicine Berlin, Germany; p Department of Radiotherapy, Medical Faculty, University of Duisburg-Essen, Essen, Germany; q Department of Radiotherapy and Oncology, Goethe-University Frankfurt, Germany; r Department of Radiation Oncology, Medical Center, Medical Faculty, University of Freiburg, Germany; s Heidelberg Institute of Radiation Oncology (HIRO), National Center for Radiation Research in Oncology (NCRO), University of Heidelberg Medical School and German Cancer Research Center (DKFZ), Germany; t Heidelberg Ion Therapy Center (HIT), Department of Radiation Oncology, University of Heidelberg Medical School, Germany; u National Center for Tumor Diseases (NCT), University of Heidelberg Medical School and German Cancer Research Center (DKFZ), Germany; v Clinical Cooperation Unit Radiation Oncology, University of Heidelberg Medical School and German Cancer Research Center (DKFZ), Germany; w Department of Radiotherapy and Radiation Oncology, University Hospital, Ludwig-Maximilians-Universität, Munich, Germany; x Clinical Cooperation Group Personalized Radiotherapy in Head and Neck Cancer, Helmholtz Zentrum Munich, Neuherberg, Germany; y Department of RadioOncology, Technische Universität München, Germany; z Department of Radiation Sciences (DRS), Institut für Innovative Radiotherapie (iRT), Helmholtz Zentrum Munich, Neuherberg, Germany; aa Department of Radiation Oncology, University Hospital Tübingen, Tübingen, Germany; ab Institute of Pathology, Faculty of Medicine and University Hospital Carl Gustav Carus, Technische Universität Dresden, Germany; ac Tumour- and Normal Tissue Bank, University Cancer Centre (UCC), University Hospital Carl Gustav Carus, Technische Universität Dresden, Germany; ad German Cancer Research Center (DKFZ), Division of Radiooncology/Radiobiology, Heidelberg, Germany

**Keywords:** Head and Neck Cancer, Gene signature, Xenograft models, Outcome Prediction, Cetuximab, EGFR

## Abstract

In a previous preclinical trial on human HNSCC xenografts, 18 genes were predictive for adding cetuximab to fractionated radiotherapy. Here, we apply the corresponding 18-gene classifier to two HNSCC patient cohorts treated with combined radiochemotherapy (without cetuximab). We show that the classifier is related to EGFR expression and stratifies patients for loco-regional control (LRC) in both cohorts.

## Highlights

• Translation of 18-gene signature from HNSCC xenografts to two HNSCC patient cohorts.
• Significant relation of the classifier to EGFR expression and LRC.
• Similarities were observed between xenograft and patient data.

## Introduction

The epidermal growth factor receptor (EGFR) represents a target in cancer therapy, in particular for head and neck squamous cell carcinoma (HNSCC) [1], where EGFR overexpression is associated with poor prognosis [2]. For patients with locally advanced HNSCC, combined irradiation and inhibition of the EGFR with a chimeric monoclonal antibody (cetuximab) is an approved treatment alternative to concurrent, cisplatin-based radiochemotherapy (RCTx) [[3], [4], [5]]. However, no advantage was observed in combination with radiotherapy or RCTx in unselected patients [3,6]. This indicates that predictive biomarkers need to be identified in order to select patients with a potential benefit of combined radiotherapy with EGFR inhibitors.

In a previous study, we analysed the effect of fractionated irradiation and additional inhibition of the EGFR in a preclinical trial using ten different human squamous cell carcinoma xenografts of the head and neck (HNSCC) [7]. Six out of 10 investigated tumour models showed a significant increase in local tumour control for the combined treatment with cetuximab and fractionated radiotherapy compared to irradiation alone. Out of the 6 responders, 3 showed EGFR gene amplification [7]. All of the responders to the combined therapy showed a significant increase in EGFR gene expression compared to non-responders. In addition, 18 genes were identified that showed a significant differential expression between responders and non-responders to the addition of cetuximab. Their expressions were anti-correlated to EGFR, i.e. a high expression was related to low EGFR gene expression and non-response. The genes were related to DNA repair, cell proliferation, epithelial-to-mesenchymal-transition (EMT) and hypoxia. It has been shown that these processes are also involved in response to cisplatin-based RCTx in unselected patient cohorts [[8], [9], [10], [11], [12]].

In this manuscript, we develop a binary classifier from the 18 genes that were differentially expressed between responders and non-responders to the addition of cetuximab to fractionated radiotherapy in the previous preclinical study [7]. Furthermore, we compare its characteristics between xenograft data (treated with radiotherapy and cetuximab) and two cohorts of unselected patients with HNSCC treated by primary or adjuvant cisplatin-based RCTx (without cetuximab). In particular, we study the relation of the classifier to EGFR expression and its prognostic value for loco-regional control (LRC).

## Methods

### Datasets

In this study, we re-use three previously reported datasets: (a) Sixty untreated xenografts of ten human HNSCC lines (Cal33, FaDu, SAS, SAT, UT-SCC-5, UT-SCC-8, UT-SCC-14, UT-SCC-15, UT-SCC-45, XF354; N = 6 for each) had been fixed in formalin and embedded in paraffine or were cryopreserved within previous local tumour control experiments [7,13]. Two patient datasets are considered, with the diagnosis of locally advanced disease: (b) Patients from the retrospective HNSCC cohort of the German Cancer Consortium − Radiation Oncology Group (DKTK-ROG) treated with primary RCTx (cisplatin- or mitomycin-C-based) between 2005 and 2011 in 6 different institutions (N = 158, median dose 72 Gy). Inclusion criteria, data collection, handling and analyses of biomaterial have been previously described [14]. (c) Patients from the retrospective HNSCC cohort of the DKTK-ROG who were treated with surgery followed by postoperative cisplatin-based RCTx (PORT-C) between 2004 and 2012 in 9 different institutions (N = 221, median dose 64 Gy). Details of this cohort have been described before [15,16].

### Gene classifier

For gene expression analysis of xenografts, 10-mm frozen cross-sections of untreated tumours were used. Total RNA was extracted according to the manufacturer‘s instructions (Qiagen, RNeasy Mini Kit), and 80 ng total RNA was used per sample [7]. For both patient cohorts, formalin-fixed, paraffin-embedded (FFPE) tissues were prepared as described previously [14,16]. Gene expression analyses were performed by nCounter technology (nanoString Technologies, Seattle, WA) using an in-house radiobiological gene panel of 209 genes. The panel differed slightly for the adjuvant patient cohort as compared to the xenografts and the primary patient cohort [12]. Details on the procedure and data processing are given in [12]. Gene expressions were available for N = 138 and N = 196 patients in the primary and adjuvant cohort, respectively. The main characteristics of these patients are given in Table 1.

**Table 1:** Patient characteristics of the cohorts treated with primary and adjuvant radiochemotherapy.

	**Primary cohort**		**Adjuvant cohort**	
**Parameter**	**Median**	**Range**	**Median**	**Range**
Age (years)	59	39–82	57	24–75
Dose (Gy)	72	68.4–74	64	56–68.4
Tumour volume (cm^3^)	27	4–176	−	−
	**Number**	**Percent**	**Number**	**Percent**
Sex (female/male)	24/114	17/83	39/157	20/80
Localisation (oral cavity/oropharynx/ hypopharynx)	23/71/44	17/51/32	56/113/27	28/58/14
T stage (1/2/3/4)	0/17/38/83	0/12/28/60	33/90/44/29	17/46/22/15
N stage (0/1/2/3)	26/5/99/8	19/3/72/6	20/27/124/25	10/14/63/13
UICC stage (2/3/4)	0/12/126	0/9/91	7/31/158	4/16/80
R status (0/1/missing)	−	−	112/83/1	57/42/1
ECE status (0/1)	−	−	89/107	45/55
p16 (negative/positive/missing)	108/21/9	78/15/7	120/72/4	61/37/2
HPV16DNA (negative/positive/ missing)	121/16/1	88/11/1	130/65/1	66/33/1

Based on the nCounter data of the xenografts, 18 genes have previously been identified that were significantly differentially expressed between xenografts responding to the addition of cetuximab to fractionated radiotherapy and non-responders [7]. The genes were associated with DNA repair (BRCA1, XRCC4, XPC), cell proliferation (RIBC2, STAT5, CENPK, BIRC5, CDKN3), EMT (SNAI1, MME, TIMP, SLC3A2, FOSL1, ITGB1) and hypoxia (ALDOC, ANKRD37, BNIP3, LDHA). Here, we combined these genes to a binary 18-gene classifier, which classifies a xenograft or patient tumour as low or high expressed with respect to these 18 genes. After z-normalising the expressions of each gene, k-means clustering was performed with two cluster centres and Euclidean distance, which stratified the samples into two classes. This procedure was repeated for every cohort due to batch effects between the cohorts. We labelled the class in which the majority of the 18 genes has a higher expression as “high” and the other class as “low”. Cluster centres are reported in Supplementary Table 1. In addition, EGFR gene expression was available from the gene panel.

### Statistical analysis

For the xenograft data, the irradiation dose required to control 50 % of the tumours (TCD50) after fractionated radiotherapy and after fractionated radiotherapy with addition of cetuximab was reported previously [7]. For patients, loco-regional control (LRC) was defined as a time-to-event endpoint, calculated from the start of RCTx until the event of a local or regional recurrence or censoring. Spearman correlation was used to analyse the correlation between gene expressions. To compare the expressions and the TCD50 between groups, the Mann-Whitney-*U* test was employed. For comparing two binary parameters, the chi-squared test was used. The association of continuous expressions on LRC was evaluated by Cox regression. The impact of the 18-gene classifier on LRC was analysed by Gray’s test, considering death as a competing risk [17]. All analyses were performed with SPSS Statistics version 29 (IBM Corporation, Armonk, NY) except for calculating the cumulative incidence function and Gray’s test, which was done in R statistics 4.4.1 (cmprsk package). p-values lower than 0.05 were considered as statistically significant.

## Results

**Fig. 1:**
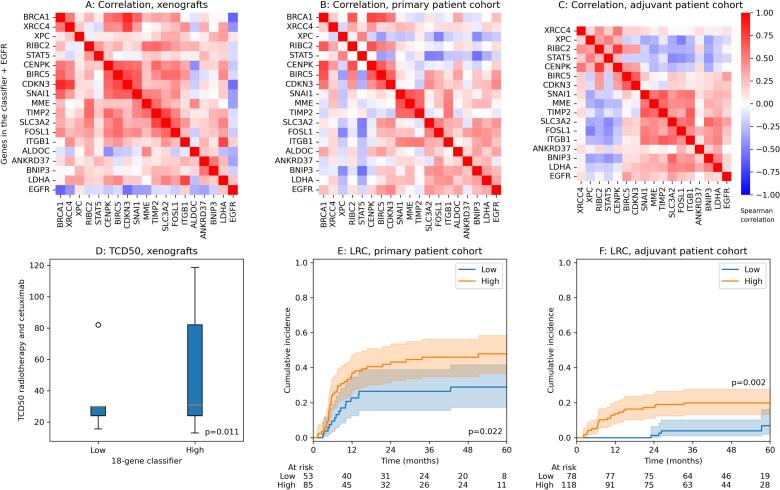
Spearman correlation between z-normalised gene expressions of genes contained in the developed classifier and EGFR expression for (A) the xenograft cohort, (B) the patient cohort treated with primary radiochemotherapy (RCTx) and (C) the patient cohort treated with adjuvant RCTx. In (D), the TCD50 after fractionated radiotherapy and cetuximab is compared between the xenograft groups defined by the 18-gene classifier. Patient stratification by the classifier for loco-regional control is shown in (E) for the primary patient cohort and (F) for the adjuvant cohort. The cumulative incidence function is shown accounting for the competing risk of death. p-values in (E) and (F) originate from Gray’s test.

For the xenografts receiving combination treatment with cetuximab, most of the 18 genes were expressed in the same direction, i.e. showed a positive Spearman correlation (98.6 % of all significant pairwise correlation coefficients), see Fig. 1A. Fifteen out of 18 genes were negatively correlated with EGFR gene expression (significant correlation for 7 of 18). After k-means clustering, the 18-gene classifier showed a statistical trend for a high expression of the 18 clustered genes to be related to low EGFR (p = 0.081). The models Cal33, SAT, UT-SCC-15 and UT-SCC-8 were mainly classified to the low expression group of the 18-gene classifier and FaDu, SAS, UT-SCCC-14, UT-SCC-45, UT-SCC-5 and XF354 to the high expression group. While EGFR gene expression was positively associated with TCD50 after radiotherapy alone (p = 0.014), the 18-gene classifier was not related to that endpoint (p = 0.68) but to the TCD50 after combined radiotherapy and cetuximab treatment (p = 0.011), Fig. 1D. Here, the high-expression group showed higher TCD50, i.e. was more resistant to the combined irradiation/cetuximab treatment. Also, the low expression group of the classifier showed a larger reduction of the TCD50 after combined radiotherapy and cetuximab compared to the subgroup with high expression of the classifier (p < 0.001). In addition, the 18-gene signature was associated with EGFR gene amplification determined by FISH (p = 0.002) [7].

For the patient cohort of the DKTK-ROG receiving primary RCTx (but without cetuximab), the expressions of STAT5 and XPC were mostly negatively correlated to the remaining genes (Fig. 1B). Eleven out of 18 individual expressions of the classifier were significantly associated with EGFR gene expression, most of them with a positive correlation (10 of 11). This positive correlation with EGFR translated to the 18-gene classifier (p = 0.005). While EGFR expression was not associated with LRC (p = 0.42), the 18-gene classifier showed a significant association (p = 0.022), with a lower LRC in the high-expression subgroup (Fig. 1E). From the clinical parameters reported in Table 1, a high expression of the classifier was significantly associated with p16 negativity, larger tumour volume, and lower age (Supplementary Table 2).

For the patient cohort of the DKTK-ROG receiving adjuvant RCTx, two genes were missing in the gene panel (ALDOC and BRCA1). The remaining 16 genes were used for determining the classifier. The expressions of CENPK, STAT5, RIBC2 and XPC were mostly negatively correlated to the remaining genes, similar to the primary cohort (Fig. 1C). Twelve out of 16 expressions were significantly associated with EGFR expression, most of them with a positive correlation (10 of 12). This positive correlation with EGFR translated to the gene classifier (p = 0.001). Higher EGFR expression was associated with lower LRC in Cox regression (p = 0.013). The classifier was significantly associated with LRC (p = 0.002), with the high-expression subgroup showing significantly more loco-regional recurrences (Fig. 1F). Several clinical parameters were related to the classifier. A high expression was significantly associated with tumour location in the oral cavity, high T stage, and p16 negativity. After correction for these related parameters in multivariable Cox regression, the classifier lost its significant association with LRC in both patient cohorts (primary RCTx: p = 0.12, adjuvant RCTx: p = 0.14).

## Discussion

In this study, we applied an 18-gene classifier, based on genes that were identified as predictive for the addition of cetuximab to fractionated radiotherapy in HNSCC xenografts, for the stratification of two patient cohorts with locally advanced HNSCC treated with RCTx but without cetuximab. In xenografts, the 18 genes were mainly anti-correlated to EGFR gene expression with respect to response to the combined treatment including irradiation plus cetuximab, i.e. higher EGFR gene expression was associated with lower expression of the 18 gene classifier. Furthermore, higher expression of the 18-gene signature was significantly associated with higher TCD50. The prognostic value of the signature for TCD50 after combination of radiotherapy and cetuximab in xenografts translated to the univariable analysis of LRC for both patient cohorts who received combined RCTx. A high expression of the gene signature was related to lower LRC but also to patient and tumour characteristics such as oral cavity carcinoma, high T stage as well as p16 negativity. Due to these associations, the gene classifier lost its significant relation with LRC in multivariable analysis.

In the two patient cohorts, a high expression of the 18-gene classifier was significantly associated with p16-negative tumours and large tumour volumes. The classifier includes genes related to DNA repair (e.g. BRCA [18]), proliferation (e.g. STAT5 [19]), epithelial-mesenchymal-transition (EMT) (e.g. SNAI1 [20]) and hypoxia [9], which are often up-regulated in more aggressive and radioresistant tumours leading to poor prognosis (e.g. large tumours, oral cavity tumours), while HPV-positive HNSCC show higher response rates to radiotherapy due to different underlying biology. However, the genes may not exclusively play a role in radioresistance but could also be involved in resistance to concomitant systemic therapy with cisplatin and/ or EGFR-inhibitors such as cetuximab. For STAT5, it has been shown, that its activation plays a role in EMT, resistance to cisplatin-mediated apoptosis as well as EGFR-inhibition [21]. Also for the EMT-marker SNAI1, its contribution to the induction of an increased cisplatin-resistance [22] as well as to increased resistance to EGFR inhibition with erlotinib [23] has been previously described. Furthermore, a positive correlation between the classifier and EGFR expression was found in the two patient cohorts consisting of both HPV-positive and HPV-negative tumours. In addition, both the classifier and the EGFR gene expression were associated with decreased LRC in patients receiving adjuvant RCTx. After primary RCTx the classifier but not the EGFR gene expression (p = 0.42) was associated with LRC. In the literature, there are conflicting data regarding EGFR expression and radioresistance. In HPV-positive HNSCC models, EGFR-induced impairment of DNA repair and higher response rates to radiotherapy were demonstrated *in vitro* and *in vivo* [24]. In contrast, another study showed that inhibition of the EGFR does not lead to increased radiosensitisation on HPV-positive HNSCC [25]. Furthermore, clinical trials with “de-escalation strategies” that replace cisplatin by cetuximab have failed [[26], [27], [28]].

However, there are limitations of our study: The 18-gene classifier originates from a differential gene expression analysis between xenografts responding to additional cetuximab treatment and non-responders. In contrast, the patient cohorts considered in this manuscript did not receive treatment with cetuximab or another EGFR inhibitor, since cetuximab-treated patient cohorts were not available to us. Therefore, the predictive value of the 18 genes could not be assessed. Furthermore, predictive biomarkers for a specific intervention are not necessarily prognostic on unselected patients. Thus, the observed association of the 18-gene classifier with LRC on both patient cohorts may therefore be considered as coincidental or caused by the preselection of genes in the nanoString panel, which was composed to include known factors of radioresistance in HNSCC, such as hypoxia, EMT or DNA repair [[8], [9], [10], [11], [12]]. These factors are important for both, patients treated with adjuvant or primary RCTx. The impact of hypoxia in patients treated with postoperative RCTx may be surprising but suggests its impact also by other radiobiology-related mechanisms, such as increased stemness and tumour invasiveness, as discussed previously [16]. Nevertheless, the prognostic value of the classifier for the combined treatment approaches (combined radiotherapy and cetuximab or combined radiochemotherapy as primary or postoperative therapy) warrants further investigation. Validation in larger datasets is required, also to investigate the relevance of the classifier in combination with other tumour characteristics, such as T stage and p16 status.

Taken together, similarities were observed between xenografts and patient datasets: For both data types, increased EGFR gene expression was associated with radioresistance, i.e. to a higher TCD50 after fractionated radiotherapy in xenografts or to lower LRC for patients. In addition, a high expression of the 18-gene classifier was consistently related to therapy resistance, i.e. a higher TCD50 after combined radiotherapy and cetuximab or lower LRC after RCTx. Importantly, there was no significant association between the 18-gene classifier and the TCD50 after irradiation alone in xenografts. This indicates that the 18 gene classifier might be more suitable to predict the response to the combined treatment (irradiation with cetuximab or combined radiochemotherapy). Further analyses are required to verify this hypothesis.

In summary, this study not only evaluated the prognostic value of biomarker results obtained from HNSCC xenografts to unselected patient data, but also the potential of xenografts as valuable models for the development of biomarkers for individualized radiotherapy [29]. The 18-gene classifier may be further investigated for the stratification of patients with locally advanced HNSCC for combined treatment approaches.

## CRediT authorship contribution statement

**Steffen Löck:** Conceptualization, Data curation, Formal analysis, Methodology, Resources, Writing – original draft, Writing – review & editing. **Lydia Koi:** Data curation, Methodology, Resources, Writing – review & editing. **Kristin Gurtner:** Data curation, Methodology, Resources, Writing – review & editing. **Fabian Lohaus:** Data curation, Methodology, Resources, Writing – review & editing. **Max Kemper:** Data curation, Methodology, Resources, Writing – review & editing. **Dominik Haim:** Data curation, Methodology, Resources, Writing – review & editing. **Inge Tinhofer:** Data curation, Resources, Writing – review & editing. **Goda Kalinauskaite:** Data curation, Resources, Writing – review & editing. **Martin Stuschke:** Data curation, Resources, Writing – review & editing. **Maximilian Fleischmann:** Data curation, Resources, Writing – review & editing. **Claus Rödel:** Data curation, Resources, Writing – review & editing. **Anca-Ligia Grosu:** Data curation, Resources, Writing – review & editing. **Jürgen Debus:** Data curation, Resources, Writing – review & editing. **Claus Belka:** Data curation, Resources, Writing – review & editing. **Stephanie Combs:** Data curation, Resources, Writing – review & editing. **Simon Boeke:** Data curation, Resources, Writing – review & editing. **Gustavo Baretton:** Data curation, Methodology, Resources, Writing – review & editing. **Michael Baumann:** Conceptualization, Data curation, Project administration, Resources, Supervision, Writing – review & editing. **Mechthild Krause:** Data curation, Conceptualization, Project administration, Resources, Supervision, Writing – review & editing. **Annett Linge:** Conceptualization, Data curation, Methodology, Resources, Supervision, Writing – original draft, Writing – review & editing.

## Declaration of competing interest

The authors declare the following financial interests/personal relationships which may be considered as potential competing interests: In the past 5 years, Dr. Mechthild Krause received funding for her research projects by Merck KGaA (2018-2020 for clinical study). Dr. Mechthild Krause and Dr. Annett Linge were involved in a publicly funded (German Federal Ministry of Education and Research) project with the companies Medipan (2019-2022), Attomol GmbH (2019-2022), GA Generic Assays GmbH (2019-2022), Gesellschaft für medizinische und wissenschaftliche genetische Analysen (2019-2022), Lipotype GmbH (2019-2022) and PolyAn GmbH (2019-2022). Dr. Krause and Dr. Linge confirm that, to the best of their knowledge, none of the above-mentioned funding sources were involved in the preparation of this paper.

Michael Baumann, CEO and Scientific Chair of the German Cancer Research Center (DKFZ, Heidelberg) is responsible for collaborations with a large number of companies and institutions worldwide. In this capacity, he has signed contracts for research funding and/or collaborations, including commercial transfers, with industry and academia on behalf of his institute(s) and staff. He is a member of several supervisory boards, advisory boards and boards of trustees. Michael Baumann confirms that he has no conflict of interest with respect to this paper.

Dr. Simon Boeke received speakers’ honoraria and travel expenses by MERCK AG.
