# Supplementary material for: Application of a preclinical 18-gene classifier to patients with locally advanced HNSCC
Source: Clin Transl Radiat Oncol. 2025 Nov 5;56:101067. doi: 10.1016/j.ctro.2025.101067 (PMC12639843; doi:10.1016/j.ctro.2025.101067)
Supplement: Supplementary Data 1 [file mmc1.pdf]

Supplementary Table 1: Cluster centres from k-means clustering for both patient cohorts.

	Adjuvant RCTx		Primary RCTx	
**Gene**	**Centre low**	**Centre high**	**Centre low**	**Centre high**
ALDOC			-0,203086	0,12663
ANKRD37	-0,0245893	0,016254	-0,306623	0,191188
BIRC5	-0,0823268	0,0544194	-0,368987	0,230074
BNIP3	-0,7017296	0,4638552	-0,718184	0,447809
BRCA1			0,155949	-0,097239
CDKN3	-0,0519069	0,0343113	-0,370018	0,230717
CENPK	0,6113486	-0,4041118	0,204642	-0,1276
FOSL1	-0,7385677	0,4882057	-0,812521	0,506631
ITGB1	-0,724194	0,4787045	-0,629626	0,39259
LDHA	-0,4952843	0,3273913	-0,650101	0,405357
MME	-0,6846834	0,4525873	-0,583682	0,363943
RIBC2	0,7121015	-0,4707112	0,232291	-0,14484
SLC3A2	-0,8558756	0,5657483	-0,640316	0,399256
SNAI1	-0,5706445	0,3772057	-0,459291	0,286382
STAT5	0,5804713	-0,3837013	0,71648	-0,446747
TIMP2	-0,5501812	0,3636791	-0,264055	0,164646
XPC	0,6245461	-0,4128355	0,66555	-0,41499
XRCC4	0,2336246	-0,1544298	-0,110163	0,06869

Supplementary Table 2: Comparison of parameters between the low and high expression group of the 18-gene signature for both patient cohorts. p-values originate from Mann-Whitney-U tests for continuous parameters and chi-squared tests for categorical parameters.

	Adjuvant RCTx			Primary RCTx		
	**Low**	**High**		**Low**	**High**	
	**Median (range) / Number**	**Median (range) / Number**	**p-value**	**Median (range) / Number**	**Median (range) / Number**	**p-value**
Age (years)	58 (29-75)	56 (24-73)	0.24	62 (44-82)	57 (39-79)	**0.034**
Dose (Gy)	64 (56-68.4)	64 (59.4-68.4)	0.29	72 (68.4-72)	72 (69.2-74)	0.65
Tumour volume (cm^3)	-	-	-	21 (5-94)	30 (4-176)	**<0.001**
Sex (female/male)	12/66	27/91	0.20	8/45	16/69	0.57
Localisation (oral cavity/oropharynx/ hypopharynx)	5/64/9	51/49/18	**<0.001**	6/29/18	17/42/26	0.41
T stage (1/2/3/4)	16/42/14/6	17/48/30/23	**0.038**	0/9/13/31	0/8/25/52	0.40
N stage (0/1/2/3)	4/9/56/9	16/18/68/16	0.15	6/2/43/2	20/3/56/6	0.24
UICC stage (2/3/4)	2/9/67	5/22/91	0.31	0/5/48	0/7/78	0.81
R status (0/1/missing)	48/29/1	64/54/0	0.26	-	-	-
ECE status (0/1)	33/45	56/62	0.48	-	-	-
p16 (negative/positive/missing)	26/50/2	94/22/2	**<0.001**	33/16/4	75/5/5	**<0.001**
HPV16DNA (negative/positive/ missing)	28/49/1	102/16/0	**<0.001**	40/13/0	81/3/1	**<0.001**
